# Unwarranted hysterectomy in a case of oro-vaginal-vulvar lichen planus in a young woman: a case report

**DOI:** 10.1186/s13256-021-02720-w

**Published:** 2021-02-26

**Authors:** Lajya Devi Goyal, Priyanka Garg, Manmeet Kaur

**Affiliations:** 1 Department of obstetrics and gynecology, All India Institute of Medical Sciences, Bathinda, Punjab 151001 India; 2 Department of Pathology, All India Institute of Medical Sciences, Bathinda, India

**Keywords:** Oro-vaginal-vulvar lichen planus, Dyspareunia, Corticosteroids, Vulva

## Abstract

**Background:**

Lichen planus is a rare autoimmune disease primarily affecting the skin and mucous membranes of the oral mucosa, vulva, and vagina. Diagnosis is difficult and often delayed as the clinicians do not associate the oral symptoms with the genital symptoms. This has a negative impact on the out-of-pocket expenditure and quality of life of the patients. We report this case, as only anecdotal cases have been reported so far from a developing country such as India. We highlight the unindicated hysterectomy that the patient had undergone because of lack of awareness regarding this condition. Our case report also highlights the importance of the multidisciplinary team approach to optimize outcomes and avoid unnecessary morbidity to such patients.

**Case presentation:**

We report a North-Indian patient with oro-vaginal-vulvar lichen planus who presented to us with complaints of recurrent vulvovaginal symptoms for the last 5 years. She had been previously treated with multiple courses of antibiotics, antifungals, and topical steroids over the course of 3 years and finally offered laparoscopic-assisted vaginal hysterectomy (LAVH) by a private practitioner but got no relief. She also had complained of oral symptoms in the form of a burning sensation after eating spicy food, but did not seek any treatment for this. After multidisciplinary team discussion, a final diagnosis of oro-vaginal-vulvar lichen planus was made at our institute based on the clinical and histopathological findings. The patient was immediately started on oral prednisolone to which she responded with improvement in her symptoms.

**Conclusion:**

Lichen planus is a chronic painful condition with significant impact on the quality of life. Women often suffer for several years before an accurate diagnosis is made. Treatment is challenging and needs to be individualized with a multidisciplinary approach to prevent progressive anatomical distortion and associated morbidity.

## Introduction

Lichen planus (LP) is a relatively rare chronic inflammatory mucocutaneous disorder with prevalence ranging between 1 and 2% [1]. It is more common in older women aged 50–60 years compared to those in the reproductive age group [1]. The exact etiology is unknown but previously published data suggest T-cell mediated autoimmunity as the most relevant explanation [2]. LP can present with varied systemic manifestations, with cutaneous lesions being the most common, followed by involvement of the mucosa, nails, and scalp. Concurrent involvement of multiple mucosal sites, referred to as “plurimucosal lichen planus,” is reported in 60–70% cases [3]. The oral cavity is the most commonly affected site with simultaneous involvement of the genitalia in about 30% of cases and occasionally the stomach, ocular and otic surfaces, and esophagus. Among the different clinical subtypes of mucosal LP, atrophic-erosive LP is the most frequent in the course of oro-vaginal-vulvar LP (OVVLP). This condition is extremely painful, resulting in irreversible scarring, and if left untreated can rarely progress to squamous cell carcinoma [4]. Currently, there are no evidence-based guidelines on the management of LP. Various medications have been tried in previously published studies with topical corticosteroids being the first-line therapeutic drug. We report this very rare case of OVVLP in a patient who was erroneously hysterectomized at a very young age under the pretext of curing this condition. Despite visiting multiple gynecologists and dermatologists, a correct diagnosis was not made because of the treating clinicians' lack of knowledge owing to rarity of this condition. We will also highlight the role of timely diagnosis and prompt management through a multidisciplinary approach, which is of utmost importance to prevent debilitating complications and prolonged agony for the patient.

## Case report

We report the case of a 32-year-old North Indian multiparous female who presented to our gynecological outpatient department in April 2020 with complaints of recurrent vulvovaginal symptoms in the form of vaginal discharge, vaginal ulceration and burning vulvar pain for the last 5 years. She had been previously treated with multiple course of antibiotics, antifungals, and topical steroids, but did not get any relief. After 3 years of unhelpful treatment, she was offered laparoscopic-assisted vaginal hysterectomy (LAVH) in February 2018, with the assurance of relief of symptoms by a private practitioner. However, the procedure did not relieve her agony, and the symptoms persisted. Histopathology of the uterus was unremarkable. After 3 months of LAVH, she presented with dyspareunia. Intercourse became increasingly difficult and finally impossible. Her previous menstrual cycles had been regular with normal amounts of bleeding. She had two living children, both delivered vaginally without any complications. She was a homemaker, non-alcoholic, and non-smoker. There was no history of intake of any indigenous medicine before the onset of the disease. Her past medical and family history was also unremarkable. Further review of the medical history disclosed that she also had complained of oral symptoms in the form of a burning sensation after eating spicy food, but did not seek any treatment for this.

**Fig. 1.**
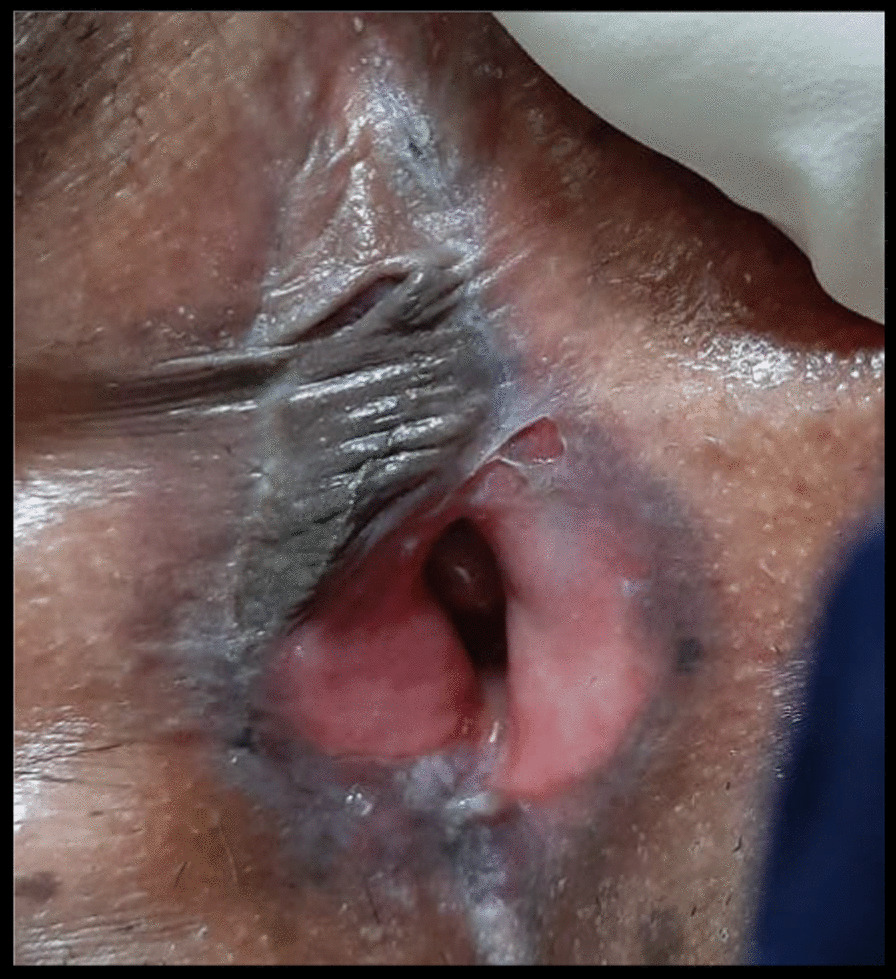
Erosive lichen planus of genitalia showing white striae and marked reduction of the vagina

**Fig. 2.**
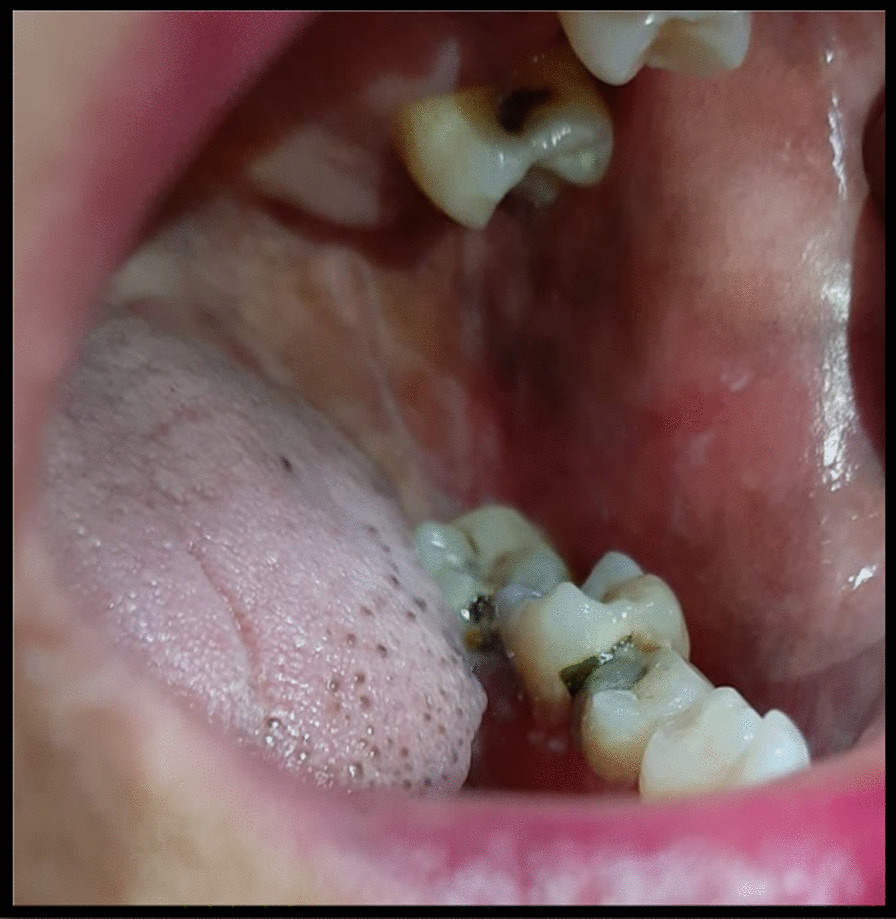
Typical Wickham striae in the posterior part of the buccal mucosa

**Fig. 3.**
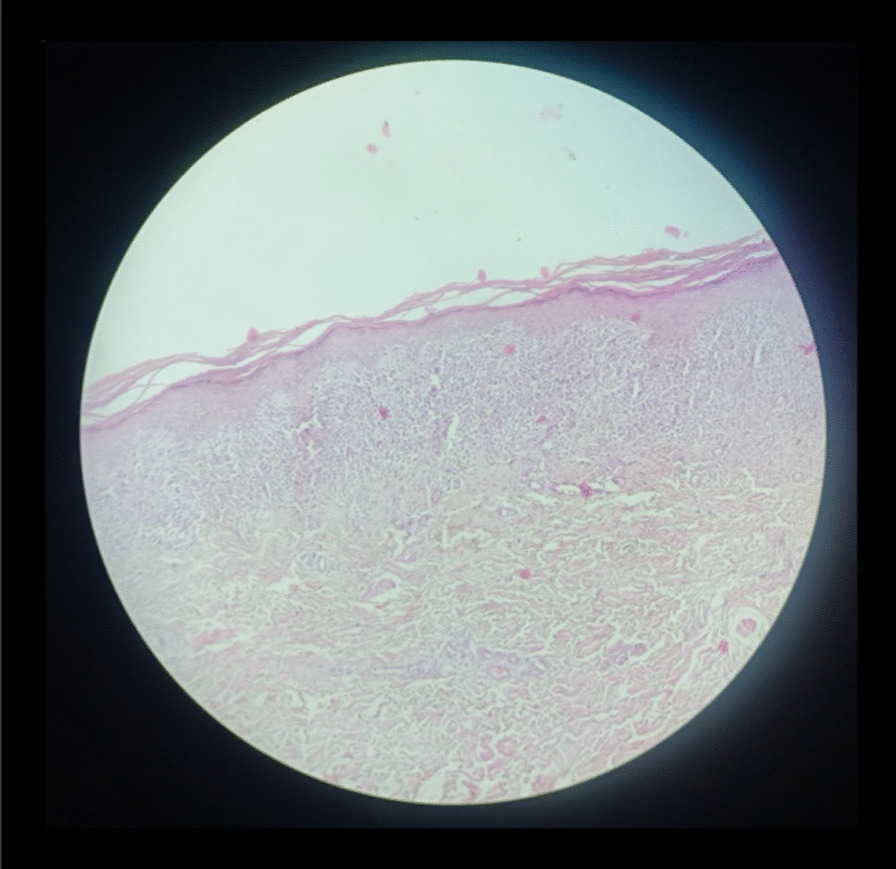
Hematoxylin and eosin-stained section (× 10) shows hyperkeratosis of the lining epithelium with a prominent granular layer and loss of rete ridges

**Fig. 4.**
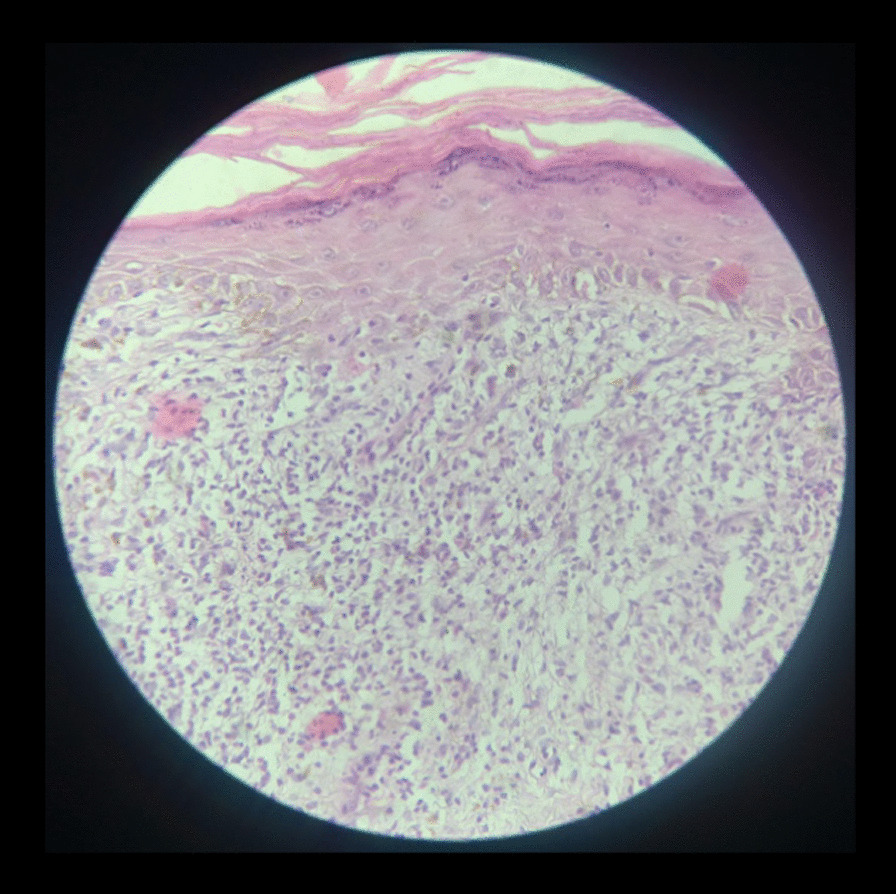
Hematoxylin and eosin-stained section (× 40) shows mainly superficial dermis infiltrated by a band-like lymphocytic infiltrate extending to the dermo-epidermal junction

On examination, she was a female of average build with excellent general health. Her blood pressure was 120/70 mmHg, pulse rate was 82/min, and she was afebrile to the touch. Systemic examination was unremarkable. On local examination, we observed that ulcers were present on the vulvovaginal mucosa involving the posterior fourchette along with white plaques on the labia minor, vaginal introitus, and surrounding vulvar skin. A vaginal speculum could not be inserted. Per vaginum examination only allowed entry of the tip of the little finger, and the area was extremely tender. Detailed examination showed that there was a significant decrease in the diameter and length of the vagina. The anterior and posterior vaginal walls were severely adherent to each other and could not be separated (Fig. 1). Oral examination revealed the presence of white plaques and erosions on the left buccal mucosa with no lesions on other parts of the oral cavity, in other words, the gingiva and throat (Fig. 2). Dermatological consultation was taken, and a provisional diagnosis of OVVLP was made. The patient was advised about the possible diagnosis, and after receiving informed written consent, vulvar and oral biopsy was taken. Preliminary blood investigations were normal. Her hepatitis B and C, antinuclear antibody tests and antithyroid antibody tests were also negative. The histopathology of vulvar biopsy revealed a hyperkeratotic, acanthotic stratified squamous epithelial lining with prominence of the granular layer. The superficial dermis mainly showed a band-like lymphocytic infiltrate (lichenoid pattern). However, acanthosis was absent in the older lesions with thinned out epithelium and loss of rete ridges (Figs. 3 and 4). Oral biopsy revealed a hyperkeratotic, acanthotic stratified squamous epithelial lining with hydropic degeneration of the basal layer. The subepithelium showed a mainly lymphocytic cell infiltrate (image not available). These findings suggested an inflammatory lesion. After multidisciplinary discussion of the case, based on the clinical picture and biopsy report, the patient was diagnosed with OVVLP ruling out lichen sclerosis and other immune bullous conditions. Since the patient had previously used topical steroids with no improvement, she was put on oral prednisolone with a starting dose of 40 mg once daily. After 4 weeks of treatment, the patient reported marked symptomatic relief of the vulvovaginal symptoms. Vaginal examination allowed one finger entry without any difficulty with improved vaginal elasticity. Her oral symptoms had also improved remarkably although mild lesions were still visible. Steroids were gradually tapered to a maintenance dose of 10 mg over 3–4 weeks. She was also advised to do vaginal dilatation at home and instructed in perineal care. On a further follow-up visit at 3 months, the patient reported normal sexual activity with only occasional mild pain. The vulvar burning sensation and oral lesions had also totally disappeared. The patient was then advised to return for follow-up every 6 months; however, she was lost to follow-up after that.

## Discussion

We report a case of oro-vaginal-vulvar lichen planus in a patient who was misdiagnosed and unnecessarily hysterectomized at a young age of 32 years. Only a few anecdotal cases have been reported in the literature so far [1, 3, 5]. Oro-genital LP is an uncommon condition affecting women at 50–60 years of age. The exact etiology is still under debate but postulated to be multifactorial including T-cell mediated autoimmunity against basal keratinocytes and genetic predisposition due to overexpression of certain HLA haplotypes [1].

Our case is unique as the patient presented at a young age of 32 years with no associated autoimmune disorder or similar family history. This unusual presentation at this young age indicates that it is not necessarily a peri- or postmenopausal disease as thought previously [6]. Atrophic-erosive OVVLP is the most common type, which manifests as erythematous lesions with lazy white borders or Wickham striae [6]. The prominent oral symptoms include burning pain, intolerance of spicy or acidic food and bleeding gums. The genital symptoms constitute dyspareunia, irritating vaginal discharge, vulvar burning sensation, and severe itching. If left untreated, it can lead to painful scarring resulting in severe anatomical distortion with loss of labia, buried clitoris, and introital and vaginal stenosis [7]. Our patient had burning pain on eating spicy food, and the pain from all the genital symptoms was especially severe during intercourse. As she presented late, her vagina was stenosed because of scarring. Based on the medical history of our patient, it was clear that her symptoms had started long ago and progressed over time, but an accurate diagnosis could not be made as gynecologists do not routinely associate genital and oral symptoms. The patient was finally diagnosed at our institute through a multidisciplinary approach.

Management of OVVLP is difficult, and the condition is usually chronic. Treatment includes drug therapy and adjuvants. Currently, there is no worldwide consensus on the correct therapeutic interventions. The treatment adopted at different institutes is based on previously published clinical case studies and case series. Topical steroids usually constitute the first line of treatment for LP. In a study conducted by Cooper *et al.* on 114 patients with histologically proven VLP, 89 were prescribed topical 0.05% clobetasol propionate ointment. Of these patients, 84 reported an improvement in symptoms, with complete remission of symptoms in 63. However, the results are not always consistent with topical steroids because of different preparations as seen in various studies, and systemic therapy may be required in 25–40% of cases [2, 8, 9]. Second-line drugs include topical immunosuppressants (tacrolimus and ciclosporin), systemic immunomodulators such as oral steroids, methotrexate, mycophenolate mofetil, minocycline, cyclosporine, and griseofulvin if the patient fails to respond to topical steroids or is having intolerable adverse effects. A few studies have reported good results with topical tacrolimus (a calcineurin inhibitor) because of its anti-inflammatory action; however, high-quality data are lacking regarding its efficacy [1]. Results with topical ciclosporin are debatable and mostly dissatisfying. Our patient did not respond to topical corticosteroids so was put on oral prednisolone, to which she responded and reported improvement in her symptoms. Similar results were seen in a retrospective study by Simpson *et al.*, where 7 out of 12 patients achieved control on oral prednisolone after failure with highly potent topical steroids [8]. Patients with LP often have associated hepatitis C infection. Our patient however had no such complication. Another concern is the rare development of SCC due to long-standing chronic inflammation. Concomitant anti-fungal drug therapy may be given to prevent super-added mycotic infections as a result of drug-induced immunosuppression. Adjuvant therapy consists of patient education, vulvar care and regular review of the medication history.

LP is a complex and chronic disease with delayed healing of lesions. It seriously impairs the quality of life of the patient. Patients should be counseled regarding the nature of the disease so that their expectations are reasonable. They should be regularly followed up at 6-month intervals to monitor for the development of SCC. They should also be taught about the correct method of perineal care. In addition, patients should be encouraged to seek psychological counseling to help relieve stress.

We acknowledge certain limitations in our case report. First, the patient could not be followed up after 3 months; hence, we cannot comment on the long-term benefits and side effects of the therapy offered to the patient. Second, we could not determine the etiology behind the LP in our case because of the lack of sophisticated diagnostic facilities in our health center.

## Conclusion

Lichen planus is a rare but debilitating condition requiring prolonged treatment. Unfortunately, diagnosis is difficult and often delayed. Owing to the resistant nature of the disease, multiple therapies may have to be tried before a satisfactory response is attained. Large-scale randomized controlled trials are required to develop specific therapeutic algorithms to improve the quality of life of the affected women and prevent malignant complications.

## Data Availability

Not applicable.
